# Social development level, digital literacy, problematic social network use and online collaborative learning in higher vocational medical students: mediating and moderating effects

**DOI:** 10.1186/s12909-026-08881-w

**Published:** 2026-03-07

**Authors:** Liu Dongdong, Liang Yuqian

**Affiliations:** 1 Teacher Education College, Suqian University, Suqian, Jiangsu Province China; 2 School of Public Administration, Sichuan University, Chengdu, Sichuan Province China; 3 Health Industry College, Sichuan Tianyi College, Deyang, Sichuan Province China

**Keywords:** Social development level, Online collaborative learning, Digital literacy, Problematic social network use, Gender, Higher vocational medical students

## Abstract

Grounded in Cognitive Strategy Theory and Compensatory Media Theory, this study investigates the mechanisms linking social development level to online collaborative learning among vocational medical students. It examines the parallel mediating roles of digital literacy and problematic social network use, alongside the moderating role of gender. A survey of 3,429 Chinese vocational medical students supported the hypothesized model. The results revealed that social development level positively predicted online collaborative learning. Furthermore, digital literacy mediated this relationship positively, whereas problematic social network use mediated it negatively, with the former effect being substantially stronger. Gender significantly moderated the indirect effect via digital literacy, with a stronger positive mediation observed among male students; however, no significant moderation was found for the pathway through problematic social network use. The findings provide an integrated theoretical account of the dual pathways shaping online collaborative learning and offer practical guidance for developing targeted educational interventions.

**Supplementary Information:**

The online version contains supplementary material available at 10.1186/s12909-026-08881-w.

## Introduction

Online collaborative learning (OCL) is defined as a process where groups of learners interact through digital platforms to co-construct knowledge and achieve shared learning goals. Students interact in the learning environment, actively learning by asking questions, discussing and sharing ideas, making collective decisions, and reflecting on their thoughts and experiences [5]. In the context of the digital transformation of education, OCL provides students with external incentives to maintain their motivation to learn [72] and enhances the cohesion of collaborative members and the interaction among learners [45, 75]. It offers real-time support to learners, and improves their sense of belonging and learning motivation [43] within an open learning environment [3]. The term “collaboration” also reflects that the core of online collaborative learning lies in the interaction among learning members. It is essentially a form of community organization and knowledge construction that is inseparable from social interaction and a social environment [19]. Studies have shown that learning interaction behavior is a key factor affecting the quality of collaborative learning [1]. OCL demonstrates unique characteristics for medical students in particular. Medical education emphasizes rigorous clinical reasoning and teamwork, and its collaborative tasks place far greater demands on information accuracy, role clarity, and professional communication skills than many other disciplines. Consequently, the collaborative and communication skills encompassed by social development level, as well as the ability to manage digital information effectively, are particularly critical for the success of OCL among medical students. The application of OCL in the educational field also faces uncertainties inherent in the learning process. In situations involving knowledge collaboration, competition, and opinion negotiation, the lack of self-management skills and self-discipline can lead to a burnout psychology characterized by difficulty in concentrating, inability to adapt to the accelerated flow of information, and digital addiction after a brief period of excitement [99]. Given the heavy academic and clinical demands on medical students, structured online collaborative learning may help sustain their engagement without exacerbating burnout [63]. Therefore, exploring the influencing factors and mechanisms of OCL for vocational medical students can not only enrich the related theories of OCL for this specific population.

### The relationship between social development level and online collaborative learning

Social development refers to the process by which individuals acquire language, knowledge, social rules, and social skills, enabling them to integrate into society and act freely in ways permitted by society [55]. In the context of OCL, a high level of social development equips students with stronger willingness to cooperate, communication skills, and the ability to resolve group conflicts—fundamental to effective collaboration [57]. Research shows that students with higher social development can adapt more quickly to new environments, actively seek help from peers, and are more proficient in teamwork [67]. Recent studies continue to support this, showing that individuals with higher social development perform better in online collaborative tasks [32]. These traits enable them to participate more effectively in OCL. Fundamentally, successful collaborative learning is a highly social process that depends on the quality of individual social participation and interaction [36].

### The concurrent mediating role of digital literacy and problematic social network use

While the social development level may directly impact an individual's online collaborative learning, focusing solely on this relationship does not explain the specific influences at play. It is necessary to consider factors related to individual online behavior and literacy to delve deeper into the underlying mechanisms [48]. The theory of compensatory media was proposed by the renowned American media theorist Levinson [52], whose core argument is that people's choice of subsequent media is a form of compensation for the functions of past media, but this functional compensation also comes with a loss of function, meaning that compensation and loss coexist. The learning methods afforded by network media compensate for the lack of interactivity in traditional print media, providing learners with a more open and expansive space for learning, but they also lead to an excessive dependence on electronic products among learners, with the phenomenon of problematic social network use among medical students being frequent [59, 95]. This also places higher demands on learners' digital literacy. Problematic social network use is an unhealthy excessive form of social media use, characterized by a lack of control over behavior, with continued engagement despite adverse life consequences [28]. Possessing good digital literacy means being better adapted to the digitalization of ways of living and working, and using digital technology services to meet real-world needs effectively. College students' digital literacy refers to a collection of digital competencies and abilities that should be possessed in the digital age. This not only manifests in the ability to use digital technology, digital resources, and digital tool platforms for learning and communication but, more importantly, in maintaining a vigilant mindset and not being overwhelmed by digital technology [54]. Therefore, as manifestations of the loss and compensation for individuals adapting to new media, both college students' digital literacy and problematic social network use may have an impact on the effectiveness of their online collaborative learning. Based on this, the study aims to explore the concurrent mediating role of digital literacy and problematic social network use between the social development level and online collaborative learning.

#### The mediating role of digital literacy

Digital literacy can be conceptualized as the ability to use information and communication technologies [41], which includes specific skills such as searching, evaluating, summarizing, analyzing, creating, and sharing information [20], as well as social skills like collaborating with others [13, 88]. Therefore, digital literacy itself demands certain social development levels from students. Relevant research has also confirmed the correlation between the two. Based on the social presence theory, studies have suggested a significant correlation between social interaction in the learning environment and students' cognitive engagement in online collaborative learning [46]. Early research also highlighted the value of social interaction, indicating that deep and sustained interaction among students can promote higher levels of learning [35, 82]. Moreover, in collaborative learning, an individual's metacognitive abilities are important, as is the group members' response to each other's contributions [89]. An intervention study on high school students demonstrated that training designed to improve cognition and strategic thinking significantly enhanced students' cognitive and social skills, which are fundamental components of digital literacy [64]. Therefore, social development may facilitate the enhancement of an individual's digital literacy.

Digital skills and the ability to collaboratively solve problems are considered essential skills for 21st-century students by educators [86], and their relationship has also been explored. In higher education, the cultivation of digital literacy leads to students demonstrating different levels of teamwork, such as sharing, collaborating, and cooperating [8]. Scholars have discussed the digital literacy of vocational education students in relation to online collaborative learning and categorized students' digital skill levels into high, medium, and low skill groups. The study found that students with low digital skills had lower satisfaction with online collaborative learning and poorer self-evaluations of the group processes involved in collaborative online tasks [61]. Therefore, digital literacy may have a positive impact on online collaborative learning. This perspective is further supported by empirical evidence from an experimental study showing that project-based learning significantly improved collaboration, critical thinking, and problem-solving skills among primary school students, suggesting that these competencies can be effectively fostered through structured collaborative pedagogy [78]. Strong evidence comes from mathematics education, where a structural equation modeling study found that 21st-century skills such as collaboration, problem-solving, and critical thinking were strongly positively correlated with student engagement and significantly mediated the impact of instructional methods on learning outcomes [76]. This indirectly suggests that digital literacy is likely a key mechanism for improving learning effectiveness in online collaborative settings.

#### The mediating role of problematic social network use

Previous studies have shown that interpersonal distress among adolescents significantly positively predicts their problematic mobile social network use [53]. There is limited empirical research directly exploring the relationship between social development and problematic social network use, but indirect evidence suggests that there may be a close association between the two. Firstly, looking at the specific behaviors of problematic social network use, such as online gaming, research indicates that social media use is not mutually exclusive [34], and emotional factors (perceived enjoyment and social interaction) are one of the reasons for participating in online games [16]. Secondly, considering the composition of social development. The structure and content of college students' social development itself encompass many aspects. Chen [18] summarized numerous studies, suggesting that an individual's social development includes behavioral, emotional, cognitive, and situational aspects. Some escapist behaviors and entertainment factors can lead individuals to become addicted to social media [31]. Impulsivity and inattention have been found to be psychological risk factors for social media use disorders [80]. Studies have shown that social media cognition has a significant predictive effect on problematic social network use, even surpassing the influence of age and gender [2]. Overall, the use of social media can promote the expression of one's opinions, thoughts, and feelings, and increase social connections to reduce loneliness [22, 70]. Moreover, problematic use of social media is also associated with psychological distress, reduced life satisfaction, and other related issues [49].

Research on the psychological and behavioral factors affecting college students' use of the internet for informal learning is scarce [17, 33, 79]. However, evidence suggests that prolonged exposure to internet technology may lead to technological stress and privacy risks, which can affect learners' effectiveness in online collaborative learning [14], while individuals' social behaviors also play an important role in online collaborative learning [100]. Therefore, problematic social network use may have a negative impact on online collaborative learning.

### The moderating role of gender

Gender, as a key demographic attribute, is often used as a basis for grouping college students in online collaborative learning [7]. Differences between males and females in behavioral patterns, communication styles, and emotional expression may influence collaborative learning outcomes [30]. Specifically related to our model, we propose that gender moderates the strength of the two mediating pathways. First, regarding the pathway through problematic social network use, research indicates that male participants tend to exhibit higher levels of social media disorder [11] and may face greater challenges in impulse control [23]. Consequently, when social development level is low, male students might be more susceptible than females to problematic use, leading to a stronger negative impact on collaborative learning. Second, regarding the pathway through digital literacy, studies have found that females are often associated with superior digital literacy [81] and creative thinking [92]. Thus, the positive effect of social development on digital literacy—and its subsequent indirect effect on collaborative learning—might be stronger for female students.

In summary, to comprehensively address the question of how social development level influences online collaborative learning and what gender differences exist, this study constructs a moderated parallel mediation model. The model explores the mechanisms through which social development affects learning outcomes via digital literacy and problematic social network use, with gender as a moderator. To systematically ground this investigation, the study is guided by an integrated theoretical framework combining Cognitive Strategy Theory, Compensatory Media Theory, and the I-PACE model, which will be detailed in the following section.

### Research hypotheses

Based on the foregoing literature review and theoretical reasoning, this study proposes a moderated parallel mediation model and tests the following hypotheses:

- H1: Social development level will positively predict the effectiveness of online collaborative learning.
- H2: Digital literacy will mediate the positive relationship between social development level and online collaborative learning effectiveness.
- H3: Problematic social network use will mediate the negative relationship between social development level and online collaborative learning effectiveness.
- H4: Gender will moderate the mediating effects proposed in H2 and H3. Specifically, the mediating effect of digital literacy (H2) will be stronger for female students, whereas the mediating effect of problematic social network use (H3) will be stronger for male students.

## Theoretical framework

This study integrates cognitive strategy theory, compensatory media theory, and I-PACE model to form the theoretical framework guiding the research. These three perspectives collectively explain the direct effect of social development on learning, its parallel mediating mechanisms, and the role of gender differences.

Firstly, according to the cognitive strategy theory, learning relies on the individual's internal cognitive organization and regulatory processes [90]. Students with high levels of social development have more mature cognitive strategies, such as organizational and precision processing strategies, which enable them to directly and effectively handle complex information in collaborative tasks, monitor team progress, and improve collaborative learning outcomes. This provides the core basis for hypothesis H1.

Secondly, social development not only produces direct effects, but also plays a role through two parallel intermediary paths, which requires complementary theories to explain. From a positive perspective, as mentioned earlier, mature cognitive strategies also help students develop higher digital literacy, namely the ability to efficiently manage and critically evaluate digital information. High digital literacy has become a key empowering tool for online collaboration, promoting learning. Therefore, cognitive strategy theory also supports hypothesis H2. From a negative pathway perspective, compensatory media theory provides the opposite view, suggesting that individuals will turn to the network to compensate for real-life deficiencies. Students with low levels of social development may be more prone to using problematic social networks to seek compensation due to insufficient real-life social skills, which can interfere with and harm collaborative learning. This provides a theoretical basis for hypothesis H3.

Finally, the I-PACE model integrates the above paths into a framework of "individual emotion cognition execution" interaction [12]. It elucidates that social development, as a key antecedent factor, can directly affect behavioral outcomes and can also lead to two different outcomes of digital literacy or problematic use by influencing different emotional cognitive mechanisms. More importantly, the model emphasizes that individual differences systematically regulate these intrinsic mechanisms, providing a deeper explanation for hypothesis H4.

In summary, this framework uses the I-PACE model as an overarching structure and organically integrates cognitive strategy theory and compensatory media theory, jointly constructing a comprehensive theoretical basis for explaining variable relationships.

## Methods

### Research design and methodology

This study employed a cross-sectional survey design with a convenience sampling method for data collection. Grounded in theories such as Cognitive Strategy Theory, the research was built upon a hypothesized moderated mediation model. It aimed to examine how social development level influences online collaborative learning through the parallel mediating roles of digital literacy and problematic social network use, and how this process is moderated by gender. The data analysis proceeded in two stages. First, we conducted confirmatory factor analysis (CFA) using Mplus 8.3 to examine the validity and reliability of the measurement models. Subsequently, the mediation and moderation effects in the structural model were tested using SPSS 25.0 and the PROCESS macro (version 4.0). PROCESS was selected due to its wide recognition for testing complex mediation and moderation models and its provision of the bias-corrected bootstrapping method, which offers more robust testing of indirect effects. The analytical procedures included tests for common method bias, correlation analysis, parallel mediation analysis using Model 4, and moderated mediation analysis using Model 58 to verify the research hypotheses.

## Research subjects

Among the higher vocational medical students who participated in the survey, 2,056 were male students (59.96%) and 1,373 were female students (40.04%); the average age was 19.45 ± 1.12 years. There were 1689 nursing students (49.26%), with a relatively high proportion of males. This gender composition aligns with recent trends toward greater gender diversity in Chinese vocational nursing education. Other majors included 875 pharmacy students (25.52%), 592 traditional Chinese medicine students (17.27%), 164 medical imaging technology students (4.78%), and 109 medical laboratory technology students (3.18%). Regarding daily internet usage, 653 students (19.04%) spent less than 3 h online, 1,743 students (50.83%) spent 4–6 h, 751 students (21.90%) spent 7–9 h, and 282 students (8.22%) spent 10 h or more. Additionally, 1,014 students (29.6%) reported holding a formal student leadership position (e.g., class monitor, student union member, or dormitory leader), which are common extracurricular roles in Chinese higher education institutions.

### Measurement tools

#### College students' social development level assessment scale

The study used the College Students' Social Development Level Assessment Scale developed by Liu [58]. As the original scale was in Chinese, it was adopted directly without modification, as it was originally developed and validated in the Chinese cultural context, ensuring its conceptual appropriateness for the present sample. This scale demonstrates good reliability and validity in the original validation study, consisting of 58 items, including the cognitive subscale (16 items, such as "I often learn about major events at home and abroad through newspapers, TV, the internet, radio, etc."), the affective subscale (22 items, such as "I feel disgusted with this society"), and the behavioral subscale (20 items, such as "The state of social customs has nothing to do with me"). The scale uses a 5-point Likert scale ("1" indicates "not at all like me" and "5" indicates "very much like me"). A higher total score indicates a better social development level.

#### College students' online collaborative learning effectiveness scale

The study used the College Students' Online Collaborative Learning Effectiveness Scale developed by Qi [74] in a master’s thesis, originally designed to assess online collaborative learning effectiveness among university students in blended learning contexts. Given its alignment with the current study’s setting and the strong reliability (Cronbach’s α = 0.929) and construct validity (KMO = 0.916, Bartlett’s test *p* < 0.001) reported in the original study, the Chinese version was adopted without modification. The questionnaire consists of 15 items across five dimensions: learning attitude and awareness (2 items, such as "Willing to accept group assignments and actively participate in group discussions and activities"), knowledge preparation (4 items, such as "Can clearly understand the social relationships in the online group and the role of each member"), interpersonal communication skills (4 items, such as "Can listen to different opinions from peers and accept their viewpoints"), human–computer interaction skills (4 items, such as "Can screen disordered information, find useful information, and form my own opinions"), and learning outcomes (1 item, "Can actively and effectively complete online collaborative tasks"). The scale uses a 5-point Likert scale ("1" indicates "very dissatisfied" and "5" indicates "very satisfied"). A higher total score indicates better individual online collaborative learning outcomes.

#### Problematic social network use scale

The study used the abbreviated version of the Social Media Disorder Scale (SMDS) developed by van den Eijnden et al. [87], which has been shown to have good internal consistency and test–retest reliability compared to the 27-item version. The original English scale was translated into Chinese through a standard translation and back-translation procedure. First, two bilingual postgraduate students in psychology independently translated the scale. The research team then discussed and synthesized a preliminary Chinese version. Subsequently, a translator, blind to the original scale and majoring in English, back-translated the Chinese version into English. The back-translated version was compared with the original to ensure semantic consistency. No cultural adaptations were made to the item content, as the construct of problematic social network use is considered to be context-invariant in its core manifestations. A pilot check confirmed the cultural appropriateness and clarity of the translated items for the target population. The scale consists of 9 items representing 9 dimensions, using a "yes/no" response format (0 = "no", 1 = "yes"), with a scoring range of 0 to 9. A cutoff score of 6 or higher indicated problematic social network use (PSNU) [10]. It should be noted that this threshold serves to identify at-risk behaviors rather than establish clinical diagnoses.

#### College students' digital literacy scale

The digital literacy of university students was assessed using the Information and Data Literacy Scale from the 'Global Framework for Digital Literacy' developed by UNESCO [85]. The original English scale underwent the translation and back-translation procedure. The items were deemed appropriate for the Chinese college student population without modification, as they measure fundamental digital competencies that are universal across academic contexts. Similarly, the translated version was confirmed to be appropriate and clear for Chinese students. The scale is part of UNESCO’ s validated global framework and has been widely applied in educational research. It consists of 3 items, such as "I can quickly, accurately, and comprehensively search for and obtain the required digital resources on the internet". The scale uses a 5-point Likert scale ("1" indicates "completely disagree" and "5" indicates "completely agree"). A higher total score indicates better digital literacy.

It is noteworthy that for the multidimensional scales the total scores were employed as indicators of their respective constructs in the main analysis. This is consistent with our focus on the constructs' overall effects and is supported by the good fit of the confirmatory factor analysis models, which validates the unidimensionality of the total score.

### Pilot testing

Prior to the formal survey, a pilot test was conducted on all scales. Fifty college students who met the inclusion criteria but did not participate in the formal survey were recruited to complete the questionnaire. The results indicated that the Cronbach's α coefficients for all scales were above 0.85, demonstrating good reliability within the target population. Based on feedback from the pilot participants, the wording of a few items was slightly refined to improve clarity and comprehensibility.

## Results

### Measurement model assessment

To validate the psychometric properties of the scales within our study sample, we conducted confirmatory factor analysis (CFA) and reliability tests using Mplus 8.3. As shown in Table 1, the CFA model fit indices for all scales met acceptable standards: the χ^2^/df ratios ranged from 1.782 to 2.291, both CFI and TLI values were above 0.95, and RMSEA values were all below 0.08, with all indices meeting or exceeding the recommended standards for good model fit [44]. All factor loadings exceeded the acceptable threshold of 0.60 [37], with the majority surpassing the ideal standard of 0.70. The average variance extracted (AVE) values ranged from 0.65 to 0.71, all well above the 0.50 threshold [27],and the composite reliability (CR values ranged from 0.88 to 0.95, all exceeding the 0.70 benchmark by a considerable margin, providing strong evidence for convergent validity. Furthermore, Cronbach's alpha coefficients for all scales were above 0.85, demonstrating good internal consistency reliability [69]. These results confirm the validity of the measurement tools in the current context and support the use of total scale scores in subsequent analyses.

**Table 1:** Results of confirmatory factor analysis and reliability tests

Scale	χ^2^/df	CFI	TLI	RMSEA	Factor Loadings Range	AVE	CR	Cronbach's α
Social Development Level	2.175	0.951	0.924	0.061	0.62–0.86	0.68	0.95	0.953
Online Collaborative Learning	1.782	0.953	0.952	0.063	0.65–0.89	0.71	0.94	0.982
Problematic Social Network Use	2.291	0.951	0.950	0.060	0.61–0.83	0.65	0.88	0.900
Digital Literacy	1.965	0.951	0.951	0.060	0.69–0.85	0.69	0.87	0.917

*AVE* Average Variance Extracted, *CR* Composite Reliability

### Common method bias test

To address potential common method bias, Harman's single-factor test was performed. The unrotated factor analysis extracted 9 factors with eigenvalues greater than 1, with the largest factor accounting for 30.05% of the variance, below the 40% threshold, indicating no severe common method bias [91].

### Correlation analysis among variables

Descriptive statistics and correlation analysis were conducted on the research variables (Table 2). The results showed that there were significant positive correlations among the social development level of vocational medical students, online collaborative learning effectiveness (*r* = 0.69,* p* < 0.001), and digital literacy (*r* = 0.61, *p* < 0.001), while these variables were negatively correlated with problematic social network use (*r* = −0.29, *p* < 0.001). Gender and student leadership role were also significantly correlated with key variables and were thus controlled in subsequent analyses.

**Table 2:** Means, standard deviations, and correlation analysis results for each variable (*N* = 3429)

Variable	*M*	*SD*	1	2	3	4	5	6
1 Gender (a)	—	—	1					
2 Student Leadership Role (b)	—	—	0.12***	1				
3 Social Development Level	10.26	1.57	−0.06**	0.09***	1			
4 Online Collaboration Learning Effectiveness	3.56	0.80	−0.04*	0.09***	0.69***	1		
5 Digital Literacy	3.53	0.82	−0.07***	0.07***	0.61***	0.83***	1	
6 Problematic Social Network Use	0.23	0.29	−0.01	0.10	−0.29***	−0.25***	−0.19***	1

^*^p < 0.05, **p < 0.01, ***p < 0.001 (the same below)

①All values are rounded to two decimal places for simplicity (the same below)

②The variables "Gender" and "Student Leadership Role" are control variables. Gender (a) is a dummy variable, with males coded as 0 and females as 1. Student Leadership Role (b) is also a dummy variable, with no role coded as 0 and a role as 1

③The mean value of "Social Development Level" ranges from 3 to 15, and the mean value of "Problematic Social Network Use" ranges from 0 to 1

### Testing the parallel mediation model

**Fig. 1:**
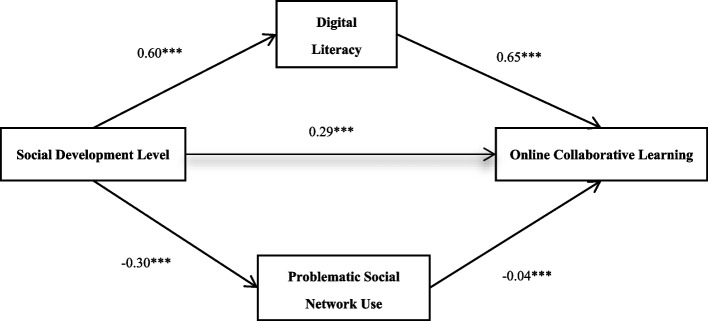
Parallel mediation effect model

Based on the correlation analysis results, significant correlations exist among the variables, and there is no issue of multicollinearity, fulfilling the prerequisites for mediation effect testing. Utilizing Model 4 of the PROCESS in SPSS developed by Hayes [38], we tested the mediation effects of digital literacy and problematic social network use between social development level and online collaboration learning, while controlling for gender and student leadership role as variables. This approach was selected because all study variables were operationalized as observed scale scores, and PROCESS provides a well-established, user-friendly framework for estimating parallel mediation effects in such contexts, without requiring the latent variable modeling that characterizes more complex structural equation modeling programs. The results are depicted in Fig. 1: Social development level significantly and positively predicts digital literacy (*β* = 0.60, *p* < 0.001) and significantly and negatively predicts problematic social network use (*β* = −0.30, *p* < 0.001). Both social development level and digital literacy significantly and positively predict online collaborative learning (*β* = 0.29, *p* < 0.001; *β* = 0.65, *p* < 0.001, respectively), while problematic social network use significantly and negatively predicts online collaboration learning (*β* = −0.04, *p* < 0.001).

Using the bias-corrected nonparametric percentile Bootstrap method to test the mediation effects, as shown in Table 3 the upper and lower limits of the bootstrap 95% confidence intervals for both mediation paths do not contain 0, indicating the presence of parallel mediation effects. The indirect effect through digital literacy is 0.39, and that through problematic social network use is 0.01.

**Table 3:** Mediation effect test (*N* = 3429)

Effect	Indirect Effect Value	Boot Standard Error	Boot CI Lower Bound	Boot CI Upper Bound
Total Effect	—	0.00	0.26	0.28
Total Mediation Effect	0.40	0.01	0.37	0.43
Digital Literacy	0.39	0.01	0.36	0.42
Problematic Social Network Use	0.01	0.00	0.01	0.02

The reported values are unstandardized effect sizes

### Testing the moderated mediation model

To further explore the reasons for gender differences in online collaborative learning outcomes, this study employed Model 58 of the SPSS macro PROCESS4.0 developed by Hayes [38], which is specifically designed to test moderating effects within a parallel mediation model (a moderated mediation model). After controlling for the variable of whether the participant held a formal student leadership position, the study tested the moderating effect of gender in the original parallel mediation model. The results, as shown in Table 4 indicate that the significance of the original paths remains consistent with previous findings. Specifically, gender has a significant moderating effect on both the first half (*β* = −0.10, *t* = −3.56, *p* < 0.01) and the second half (*β* = −0.05, *t* = −2.84, *p* < 0.05) of the mediation process involving digital literacy. However, gender does not have a significant moderating effect on either half of the mediation process involving problematic social network use.

**Table 4:** Moderated parallel mediation effect test (*N* = 3429)

Regression Equation		Overall Fitting Index			Regression Coefficient and Significance	
Outcome Variable	Predictor Variable	***R***	***R***^***2***^	***F***	***β***	***t***
Digital Literacy		0.61	0.37	503.24***		
	gender				−0.09	−3.13**
	Formal Student Leadership Position				0.04	1.19
	Social Development Level				0.60	43.43***
	gender × Social Development Level				−0.10	−3.56**
Problematic Social Network		0.30	0.09	83.66***		
	gender				−0.06	−1.77
	Formal Student Leadership Position				0.10	2.78**
	Social Development Level				−0.30	−18.19***
	gender × Social Development Level				−0.01	−0.27
Online Collaborative Learning		0.87	0.75	1395.22***		
	gender				0.04	1.97*
	Formal Student Leadership Position				0.03	1.42
	Social Development Level				0.29	25.00***
	Digital Literacy				0.65	58.89***
	Problematic Social Network				−0.04	−4.50***
	gender × Digital Literacy				−0.05	−2.84*
	gender × Problematic Social Network				−0.03	−1.53
	gender × Social Development Level				−0.03	−1.03

All variables in the model are input into the regression equation using standardized variables

**Fig. 2:**
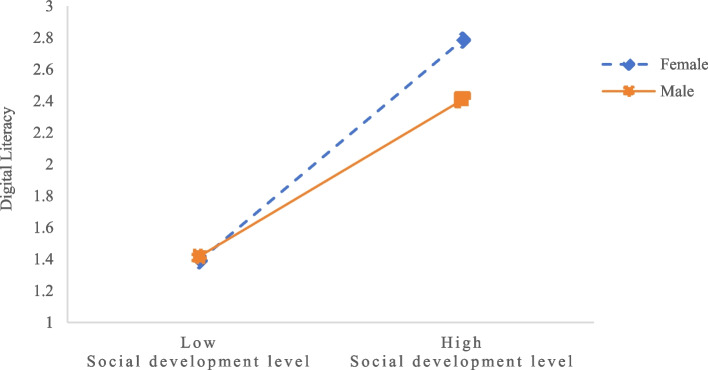
The moderating effect of gender on the relationship between social development level and digital literacy

Figure 2 illustrates the results of simple slope analysis for the first-stage mediation path (i.e., the effect of social development on digital literacy) at high (M + 1SD) and low (M-1SD) levels of social development. For females, the positive predictive effect of social development on digital literacy is significant (*β*_simple_ = 0.54, *t* = 23.30, *p* < 0.001). For males, this positive predictive effect is slightly larger (*β*_simple_ = 0.64, *t* = 37.67, *p* < 0.001).

**Fig. 3:**
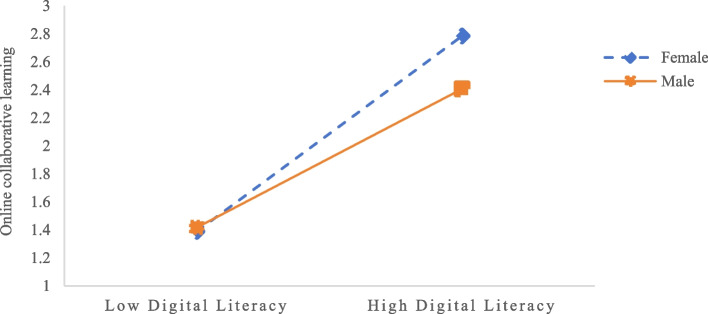
The moderating effect of gender on the relationship between digital literacy and online collaborative learning

Figure 3 displays the results of simple slope analysis for the second-stage mediation path (i.e., the effect of digital literacy on online collaborative learning outcomes) at high (M + 1SD) and low (M-1SD) levels of digital literacy. For females, the positive predictive effect of digital literacy on online collaboration learning outcomes is significant (*β*_simple_ = 0.61, *t* = 38.72, *p* < 0.001). For males, this positive predictive effect is marginally higher (*β*_simple_ = 0.67, *t* = 51.03, *p* < 0.001).

Overall, the influence of social development level on online collaboration learning outcomes through digital literacy is moderated by gender, with an index of −0.10, *SE* = 0.02, and a 95% confidence interval of [−0.14, −0.05]. For males, the positive mediation effect of digital literacy is significant, with *ab* = 0.42, *SE* = 0.02, and a 95% confidence interval of [0.39, 0.46]. For females, the positive mediation effect of digital literacy is also significant, with *ab* = 0.33, *SE* = 0.02, and a 95% confidence interval of [0.29, 0.37].

**Fig. 4:**
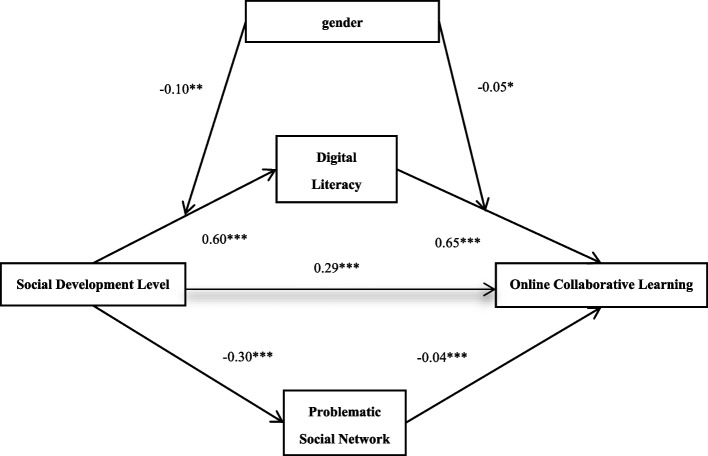
A parallel mediation model

At this juncture, Hypotheses H1, H2, and H3 have been substantiated. Simultaneously, within the framework of Hypothesis H4, it has been confirmed that in the relationship between the social development levels and online collaborative learning, digital literacy functions as a mediating variable, while gender operates as a moderating variable. Consequently, this study validates a moderated parallel mediation model, as depicted in Fig. 4.

## Discussion

This study integrates cognitive strategy theory and compensatory media theory to explore the impact of social development, as a significant developmental factor, on online collaborative learning among vocational medical students. It uncovers the mechanisms through which the social development level influences online collaborative learning, mediated by digital literacy and problematic social network use, as well as the gender differences involved. The research findings address how the social development level affects online collaborative learning and what gender differences exist, thereby possessing significant theoretical and practical value for enhancing online collaborative learning.

### Direct effects of social development level and the positive mediating role of digital literacy

This study found that the social development level not only directly and positively predicts online collaborative learning but also positively predicts it through digital literacy, aligning with the study's hypotheses. This suggests that individual cognition, social skills, and other factors may play a positive role in the development of information and communication technology (ICT) competencies, thereby facilitating online collaborative learning, which is consistent with the perspective of cognitive strategy. Students are expected to collaborate with peers and teachers through online learning platforms, coordinating learning tasks, communicating with peers and teachers, searching for learning resources, solving problems, leading learning groups, and a series of other tasks to maximize learning effectiveness [97]. In this process, individual cognitive and social adaptability play important roles. Yao et al. [96] posited that the pivotal factor exerting an influence on the sustained utilization of online learning is students' self-awareness, which is intricately intertwined with their capacity to adapt to online learning environments. In the earlier theoretical investigations, it was expounded that students' adaptability to online learning is influenced by technological elements, the learning milieu, and students' metacognitive factors, namely motivation, self-efficacy, and volition [15]. Online interactive learning engenders a distinctive learning context, wherein learning objectives, interpersonal dynamics, and emotional aspects assume equal significance owing to its "virtual" nature [21]. At the same time, recent empirical studies also have shown that online learners with strong self-regulatory abilities have more positive attitudes [101], and interaction and social regulation (the combination of self-regulation and learning strategy communication), improved social and cognitive engagement [97] can also enhance students' online learning achievements. Students with greater self-regulation and engagement demonstrate higher digital literacy and often have more motivation and interest in online or blended learning [97]. Therefore, higher vocational medical students, by enhancing their own social development and promoting their social adaptability, social skills, and cognitive engagement, can improve the effectiveness of their online collaborative learning.

Furthermore, from the perspective of the requirements of digital literacy itself, possessing digital literacy entails more than just the ability to use software or operate digital devices; it also includes complex skills such as cognition and socio-emotional skills [25]. Therefore, vocational medical students with a high sociability development level possess better learning cognitive strategies, which will promote the enhancement of their digital literacy and further result in outstanding performance in online collaborative learning. Currently, given that social media participation offers numerous learning opportunities, many interdisciplinary researchers and stakeholders in education and health advocate for conceptualizing digital participation as a fundamental right for vocational medical students. Multiple randomized controlled trials have demonstrated that collaborative learning within digital learning environments can bolster medical students' interaction and collaboration skills, problem—solving capabilities, as well as their learning satisfaction and motivation [65]. In the course of collaborative interaction, vocational medical students can develop the skills and knowledge they need to effectively interact with social media and determine which information is useful, misleading, or credible [60, 84], which will benefit their participation in online learning.

### The negative mediating role of problematic social network use

Simultaneously, this study also found that problematic social network use plays a significant negative mediating role between the social development level and online collaborative learning, aligning with the study's hypotheses. This aligns with the I-PACE model [12], which proposes that individuals with limited social or emotional resources may resort to excessive online engagement as a form of compensation, inadvertently disrupting goal-directed learning processes. Although this pathway is significant, its indirect effect is very small in magnitude, indicating limited practical relevance compared to other mechanisms in the model.

### The concurrent mediating roles of digital literacy and problematic social network use

This study further reveals that digital literacy and problematic social network use play contrasting mediating roles, one positive and the other negative, indicating their relative independence and simultaneous operation, with the indirect effect of problematic social network use being substantially smaller than that of digital literacy. Based on compensatory media theory, some scholars argue that learning in the context of digital media technology is a form of "human–machine" embodied learning, characterized by increased virtuality of the body and heightened immersion in learning, which enhances autonomy, agency, and interactivity in the learning process. However, due to insufficient alignment between top-level planning and actual learning needs, the resulting data islands further exacerbate phenomena such as knowledge islands and cognitive islands [94]. Furthermore, in terms of effect sizes, the positive mediating effect of digital literacy is greater than the negative mediating effect of problematic social network use, and the direct effect of social development level, with the total effect being significant. Specifically, the social development levels among college students positively predict digital literacy and negatively predicts problematic social network use, consistent with previous empirical research findings [9, 51]. Similarly, this relationship also applies to the connections between digital literacy and problematic social network use with online collaborative learning. Moreover, digital literacy is comparatively more closely related to online collaborative learning. The practical implications of these contrasting parallel mediators are substantial. The stronger positive mediating effect of digital literacy suggests that educational investments in boosting students' digital skills are likely to yield greater returns for collaborative learning than solely mitigating problematic social media use. This provides a clear, actionable priority for educators and institutions. In other words, the development of cognitive, social skills, and emotions among vocational medical students facilitates online collaborative learning, and the cultivation of digital literacy plays a more crucial role in enhancing their interactive and learning behaviors, thereby improving the effectiveness of online collaborative learning.

### The moderating role of gender

While the moderating role of gender in the digital literacy pathway was supported, this effect contrary to part of Hypothesis H4. Specifically, our results indicate that the mediating effect of digital literacy is stronger for male students than female students. Its moderating effect on the pathway through problematic social network use was not statistically significant. This suggests that for students with lower social development, both males and females may be equally susceptible to problematic use as a way of coping with real-world difficulties [62], leading to minimal gender differences in this specific mechanism.

In the moderating effect of gender on the mediating pathway of digital literacy, regarding the antecedent path, the positive predictive effect of social development level on digital literacy is stronger for males, potentially due to gender differences in social interaction forms and social cognition. Social interactions encompass both verbal and non-verbal exchanges [83]. In terms of non-verbal interactions, Ali et al. [4] found that female students tend to engage more in eye contact during conversations, while male students prefer not to look at their interlocutors. In terms of social cognition, evidence suggests that males and females employ different strategies in processing social information, with females being perceived as stronger empathizers and males as stronger systemizers [6]. Consequently, males may exhibit greater adaptability in online learning. Thus, when social development levels are low, males and females exhibit similar levels of digital literacy; however, as social development levels increase, both males' and females' digital literacy rises to higher levels, with males demonstrating higher digital literacy than females.

Concerning the consequent path, the positive predictive effect of digital literacy on online collaborative learning is stronger for males, potentially due to gender differences in spatial abilities and creative thinking. In terms of spatial abilities, males outperform females in both large-scale spatial abilities (the ability to cognitively process spatial information in large-scale environments; [40]) and small-scale spatial abilities (primarily involving spatial visualization and spatial relations, [42, 98]. Therefore, when digital literacy levels are low, males score slightly higher in online collaborative learning due to their advantage in spatial abilities. However, creativity test results indicate that females excel in the comprehensiveness and depth of their thinking, while males surpass females in boundary-breaking thinking [39]. Consequently, as digital literacy improves, the difference in online collaborative learning outcomes between males and females gradually diminishes. Overall, the positive mediating effect of digital literacy is stronger among males, leading to better online collaborative learning outcomes for them, consistent with previous research findings [50, 71]. Thus, although gender moderated the digital literacy mediation pathway, the pattern did not support the specific directional prediction in H4.

In summary, while females are more sensitive [56] and prefer to build relationships and collaborate [66], factors that facilitate online collaborative learning, males in this sample appeared to benefit more from the digital literacy mediation pathway, possibly due to their advantages in cognitive and spatial processing within technology-mediated contexts.

## Research significance

This study explores the influence mechanisms of online collaborative learning through an integrated theoretical lens of cognitive strategy theory and compensatory media theory, framed within the comprehensive I-PACE model. It reveals that factors at the individual socio-psychological level (such as the social development level) can exert different impacts on online collaborative learning through digital literacy and problematic social network use. Firstly, this research provides an integrated theoretical account that enriches the field of online collaborative learning. It demonstrates how the perspectives of cognitive strategy theory and compensatory media theory can be coherently framed within the I-PACE model to explain distinct pathways of influence. Future research could explore the interaction between situationally induced factors and individuals' problematic social network use from the perspective of integrating individual and situational factors. Secondly, this study finds gender differences in the mediating role of digital literacy between the social development level and online collaborative learning. Future research can investigate individual digital literacy and online collaborative learning from the perspectives of gender and gender matching. Furthermore, this study holds significant practical implications for enhancing the effectiveness of online collaborative learning in vocational education. Based on the findings, interventions should be implemented separately targeting digital literacy and problematic social network use. Given the positive effects of social development level and digital literacy, emphasis should be placed on interactions among students, between students and teachers, and between students and digital environments, to promote the development of social interaction skills and create a positive digital literacy atmosphere. Meanwhile, digital literacy education should be integrated into various majors, with targeted digital skills training based on the characteristics of each major. On this basis, it is also necessary to strengthen cybersecurity and ethics education, equipping students with basic cybersecurity awareness and skills, fostering rational expression, and respecting others' privacy and intellectual property. According to the negative effects of problematic social network use, individuals can be guided to learn to "face" problems rather than "avoid" them [29], and mindfulness-based cognitive training can be implemented based on the influencing factors of problematic social network use, which has a positive effect on alleviating such problematic use [47]. Therefore, to improve the effectiveness of online collaborative learning among higher vocational medical students, they not only need to maintain curiosity and continuously update their digital knowledge, but also enhance their ability to distinguish digital information, break free from the limitations of the "information cocoon," rationally utilize the Internet, and self—regulate their online behavior [93]. Teachers and schools should also make rational use of these findings. When organizing online collaborative learning activities, they should establish precise information—based teaching concepts and explore the development of three—dimensional training materials and teaching resources. This aligns with recent calls for enhancing teacher professional development in digital and interdisciplinary pedagogies, particularly in STEM education, where sustained, targeted training has been shown to improve teachers’ technological proficiency and instructional design capabilities [77]. Additionally, when designing teaching methods to enhance online collaborative learning for vocational students, gender factors can be considered, with collaborative learning groups established through gender pairing [26].

The findings of this study demonstrate that social development promotes online collaborative learning through digital literacy, a process grounded in concrete skills such as critical evaluation of information, coordination in group tasks, and ethical communication in digital environments. As artificial intelligence becomes increasingly integrated into educational practices, effective interaction with AI tools appears to rely on these same social-cognitive capacities. Recent evidence indicates that students’ experiences in human-to-human collaborative learning shape how they provide feedback, negotiate roles, and co-construct knowledge when working with large language models [24]. Scholars have further argued that responsible engagement with AI systems requires learners to exercise judgment, maintain agency, and reflect critically on algorithmically generated content, all of which represent competencies fundamentally nurtured through foundational digital literacy [68]. In light of these developments, the mediating role of digital literacy identified in this study may become even more critical, precisely because the integration of advanced technologies underscores the enduring value of human-centered collaborative capacities.

## Research limitations and future directions

This study has achieved the expected results, yet it is not devoid of limitations. First, the cross-sectional design prevents the determination of causal relationships among the variables. Secondly, the generalizability of the findings may be limited by the relatively homogeneous sample, which was drawn exclusively from a specific student population (vocational medical students in Sichuan Province). Furthermore, the questionnaires used in the study rely on self-reporting, which may compromise the accuracy of the assessments. Future research should employ alternative methods to further verify the causal relationships identified. Additionally, the use of total scores for multidimensional constructs may mask the unique effects of their specific dimensions. The measurement of digital literacy relied on a three-item scale, which may not fully capture the multidimensionality of this construct. Fourthly, problematic social network use is context-dependent, and the questionnaire method is unable to capture the dynamic nature of its changes. Lastly, the framework of digital literacy varies across different geographical and cultural contexts, academic disciplines, and student learning stages.

In light of limitations, future research should: (1) employ longitudinal or experimental designs to verify causality and dynamic processes; (2) validate the findings with more diverse and representative samples; (3) incorporate objective data to complement self-reported measures; and (4) investigate the distinct roles of subdimensions within broader constructs.

## Conclusion

In summary, this study examined the relationship between the social development level and online collaborative learning among vocational students, as well as the concurrent mediating roles of digital literacy and problematic social network use, while also testing the moderating role of gender. The core innovation of this research lies in revealing two distinct mediating mechanisms: the positive “facilitating” role of digital literacy versus the negative "inhibiting" role of problematic social network use. This discovery deepens our understanding of the dual pathways through which social development influences learning outcomes. The results indicate that: (1) There is a significant positive correlation between the social development level and online collaborative learning among vocational students. (2) Both digital literacy and problematic social network use mediate the relationship between the social development level and online collaborative learning among vocational students, with the positive mediating effect of digital literacy outweighing the negative mediating effect of problematic social network use. (3) Gender moderates both the antecedent and consequent paths of digital literacy, specifically, the positive mediating effect of digital literacy is stronger for male students compared to female students.

Based on these findings, this study proposes the following practical implications. First, educators should construct "digital literacy scaffolds" that deeply integrate digital tool training into collaborative tasks to help students translate competence into outcomes and the "synchronous cultivation of social-emotional skills" through high-interaction, empathetic tasks is essential [102]. Second, institutions could implement a "positive behavior support system," using clear goals and feedback to guide students toward learning tasks and mitigate the risk of problematic use [73]. In conclusion, an integrated strategy of "empowerment through literacy, guidance of behavior, and integration of emotion" provides a clear pathway for optimizing online collaborative learning.

Therefore, the hypotheses related to the social development level, problematic social network use, digital literacy, and online collaborative learning among vocational students are partially accepted. Attention should be given to the social development of vocational students, monitoring their social network use, enhancing their digital literacy, and providing professional educational guidance to continuously improve the effectiveness of online collaborative learning among vocational students.

## Supplementary Information

Supplementary Material 1.

Supplementary Material 2.

## Data Availability

Due to ethical restrictions, the raw data cannot be made publicly available. De-identified datasets are available from the corresponding author upon reasonable request, subject to approval by the Scientific Research Office of Sichuan Tianyi College Ethics Committee.
