# Supplementary material for: Social development level, digital literacy, problematic social network use and online collaborative learning in higher vocational medical students: mediating and moderating effects
Source: BMC Med Educ. 2026 Mar 7;26:614. doi: 10.1186/s12909-026-08881-w (PMC13081508; doi:10.1186/s12909-026-08881-w)
Supplement: Supplementary file 1 — Supplementary Material 1. [file 12909_2026_8881_MOESM1_ESM.pdf]

Ethical Compliance Statement

This study was conducted in accordance with the ethical principles of the World Medical Association's Declaration of Helsinki. The research protocol received approval from the Scientific Research Offce of Sichuan Tianyi College Institutional Review Board/Ethics Committee. All participants provided written informed consent after receiving detailed explanations about the study objectives, procedures, potential risks, and their right to withdraw at any time without penalty. Confidentiality of all personal data was maintained through anonymization and secure storage protocols.
