# Supplementary material for: Social development level, digital literacy, problematic social network use and online collaborative learning in higher vocational medical students: mediating and moderating effects
Source: BMC Med Educ. 2026 Mar 7;26:614. doi: 10.1186/s12909-026-08881-w (PMC13081508; doi:10.1186/s12909-026-08881-w)
Supplement: Supplementary file 2 — Supplementary Material 2. [file 12909_2026_8881_MOESM2_ESM.pdf]

Ethics Committee of Sichuan Tianyi College

Ethical Review Approval

Project Name: Research on Mental Health and Social Development of Vocational College Students against the Backdrop of Educational Digital Transformation

Project Leader: Liang Yuqian

Application Date: March 10, 2024

Approval Date: June 8, 2024

Dear Project Leader,

After rigorous examination and discussion by our Ethics Committee, we are delighted to inform you that your project, "Research on Mental Health and Social Development of Vocational College Students against the Backdrop of Educational Digital Transformation," has been officially approved by the Committee. Below are the relevant opinions of this approval:

Clear Research Purpose: This project aims to explore the specific impacts of educational digital transformation on the mental health and social development of vocational college students, possessing clear academic value and practical significance.

Adequate Protection Measures for Research Subjects: In the research, you have formulated detailed measures to protect student privacy, ensuring that the personal information security of participants remains inviolable.

Comprehensive Informed Consent Procedures: You have provided clear and understandable informed consent forms, fully informing participants of the research's purpose, process, potential risks, and their rights, which meets ethical requirements.

Reasonable Data Collection and Analysis Methods: You will adopt questionnaire surveys to collect data and employ scientific data analysis techniques to draw conclusions, ensuring the credibility and validity of the research results.

High Expected Value of Research Outcomes: The findings of this research are expected to provide theoretical foundations and practical guidance for the reform of vocational education in China, promoting the comprehensive development of students.

扫描全能王 创建

Hereby, we remind you to strictly abide by ethical norms, respect and protect the rights and interests of participants, and ensure the transparency and fairness of the entire research process. Additionally, please submit regular progress reports to us for subsequent supervision and management.

Finally, thank you for your support and cooperation with our ethical work. We wish your project every success!

Scientific Research Office of Sichuan Tianyi College

June 8, 2024

扫描全能王 创建
